# Risk-adjustment of diabetes health outcomes improves the accuracy of performance benchmarking

**DOI:** 10.1038/s41598-018-28101-w

**Published:** 2018-07-06

**Authors:** Eleanor Danek, Arul Earnest, Natalie Wischer, Sofianos Andrikopoulos, Anthony Pease, Natalie Nanayakkara, Sophia Zoungas

**Affiliations:** 1 Monash University, School of Public Health and Preventive Medicine, Melbourne, 3004 Australia; 2 Monash University, Biostatistics Unit and Registry Unit in the Department of Epidemiology and Preventive Medicine, Melbourne, 3004 Australia; 3 University of Melbourne, Islet Biology and Metabolism Research Group in the Department of Medicine, Melbourne Biostatistics Unit in the Department of Epidemiology and Preventive Medicine, Melbourne, 3010 Australia; 4 Monash University, Division of Metabolism, Ageing and Genomics in the Department of Epidemiology and Preventive Medicine, Melbourne, 3004 Australia; 5 Monash University, Diabetes, Vascular Health and Ageing, Division of Metabolism, Ageing and Genomics, School of Public Health and Preventive Medicine, Melbourne, 3004 Australia

## Abstract

Benchmarking clinical performance by comparing diabetes health outcomes across healthcare providers drives quality improvement. Non-care related patient risk factors are likely to confound clinical performance, but few studies have tested this. This cross-sectional study is the first Australian investigation to analyse the effect of risk-adjustment for non-care related patient factors on benchmarking. Data from 4,670 patients with type 2 (n = 3,496) or type 1 (n = 1,174) were analysed across 49 diabetes centres. Diabetes health outcomes (HbA1c levels, LDL-cholesterol levels, systolic blood pressure and rates of severe hypoglycaemia) were risk-adjusted for non-care related patient factors using multivariate stepwise linear and logistic regression models. Unadjusted and risk-adjusted funnel plots were constructed for each outcome to identify low-performing and high-performing outliers. Unadjusted funnel plots identified 27 low-performing outliers and 15 high-performing outliers across all diabetes health outcomes. After risk-adjustment, 22 (81%) low-performing outliers and 13 (87%) high-performing outliers became inliers. Additionally, one inlier became a low-performing outlier. Risk-adjustment of diabetes health outcomes significantly reduced false positives and false negatives for outlier performance, hence providing more accurate information to guide quality improvement activity.

## Introduction

Benchmarking of clinical performance has become a popular tool to guide quality improvement in diabetes care^1–4^. This process involves collecting data on performance indicators from different care providers. Pooled data is analysed and used to generate benchmarking reports, in which care providers are evaluated against peer performance or against a recommended standard^5,6^. Benchmarked feedback drives quality improvement by alerting care providers to areas for improvement and by stimulating healthy competition^5,7^.

Performance indicators for benchmarking should accurately reflect quality of care^8^. Examples include measures of process, such as prescribing rates, and measures of outcome, such as glycaemic control^9,10^. Outcome measures are considered the ‘gold standard’^11^ for benchmarking clinical performance in healthcare as they hold ‘intrinsic interest’^9^ for patients and clinicians. However, there are limitations to using outcomes measures to benchmark clinical performance. Variation in patient outcomes may be confounded by variables beyond the control of healthcare providers, such as age, sex and disease severity^9^. Consequently, the use of crude outcome measures for benchmarking may result in inaccurate depictions of clinical performance^12^. This may hinder the ability of healthcare providers to identify and address issues that fall under the influence of healthcare intervention.

Diabetes health outcomes are collected for benchmarking purposes in many countries including Australia^13^, the US^2,14,15^ and throughout Europe^4,16–18^. To date, benchmarking in diabetes care has been based on unadjusted diabetes health outcomes^13,14,16,18^.

Risk-adjustment for the effects of non-care related patient risk factors may improve the accuracy and fairness of benchmarking^12,19^. However, few studies have formally analysed the impact of risk-adjustment on benchmarking in diabetes care, with conflicting results^20–25^. Furthermore, few studies have used funnel plots to account for the effects of variation in patient volume on diabetes health outcomes. As a result, the extent to which risk-adjustment may improve the accuracy of benchmarking for diabetes health outcomes is unknown.

Using data obtained from the 2015 Australian National Diabetes Audit, this study sought to formally analyse the impact of risk-adjustment on diabetes benchmarking. We hypothesised that risk-adjustment reduces false positives and false negatives for outlier performance. Ultimately, the objective of our study was to inform practice in diabetes benchmarking to facilitate more accurate feedback, enabling diabetes centres to better identify and address issues in clinical care.

## Methods

### Data collection

The Australian National Diabetes Audit (ANDA) is a national quality initiative that occurs on an annual basis. Participation is voluntary and open to all centres registered with the National Association of Diabetes Centres (NADC) and other interested primary care, community-based or specialist healthcare providers in private practice^13^. Formal invitations to participate in the 2015 ANDA were issued to all eligible centres. Each participating centre was allocated a unique code to allow data collection, handling and analysis to proceed in a double-blind fashion.

Clinicians at participating centres collected de-identified patient-level data using the standardised ANDA case record forms. These case record forms utilise the minimum dataset developed by the National Diabetes Outcomes Quality Review Initiative^13^. Data items relating to patient demographics, disease characteristics, management factors and outcomes are captured. Standardised definitions for each data item were made accessible to audit-participating centres. Additionally, centres were given specific direction on how to conduct and train staff to complete the audit.

The audit period was restricted to four weeks in 2015 (either over the month of May or June), during which time each centre was required to complete a case record form for all consecutive patients with diabetes attending the diabetes centre. Diabetes case ascertainment was based on a previous or new diagnosis of diabetes, in accordance with Australian diagnostic guidelines^26^. Informed consent was obtained from all study participants. A minimum of 30 completed case-record forms, corresponding to 30 consecutive patients, was required per centre (with a two-week extension of the audit period granted to centres that were unable to collect data on 30 patients within the initial audit period).

Upon completion of the audit period, de-identified patient information was forwarded by each centre to the ANDA data management centre for review of data completeness and correctness. Duplicate entries (where multiple case records for a single patient were submitted due to multiple visits during the audit month) were handled by retaining only the most recent entry. Based on pre-determined data validation rules, lists of missing or potentially invalid data were generated and sent to participating centres to provide them with an opportunity to improve their data. Centres were encouraged to comprehensively address data queries prior to resubmission to the data management centre.

Ethics approval to undertake this study was obtained from the Monash Health Human Research Ethics Panel. All methods were performed in accordance with the relevant guidelines and regulations.

### Variables

Patient factors considered for the statistical modelling exercise were: age, sex, duration of disease, severity of disease, body mass index (BMI), country of birth and smoking history (ever smoker vs never smoker). These factors were selected based on feasibility, conceptual reasoning and clinical validity. Feasibility was determined by whether the variable was able to be retrieved/calculated from the 2015 ANDA dataset. In selecting patient factors, we referred to the conceptual framework proposed by a previous study of risk factors for glycaemic control in patients with T2DM^25^. In accordance with this framework, six domains of risk were considered: demographics, access to care, health-seeking behaviour, geographic location, disease characteristics and comorbidity. Feasible patient factors relating to one or more of these risk domains were selected and assessed for clinical face validity with the input of an experienced endocrinologist.

Severity of disease was defined using a pre-validated tool, the Diabetes Complications Severity Index^27^. Smoking history was defined as positive for ‘ever smokers’ (current or past smokers) and negative for never smokers. The decision to categorise past and current smokers as ‘ever smokers’, rather than keeping these categories separate, was made a priori. This was due to a lack of data on the duration of abstinence for past smokers.

The health outcomes considered in this study were glycated haemoglobin (HbA1c, %), low-density lipoprotein cholesterol (LDL-Ch, mmol/L), systolic blood pressure (mmHg) and the incidence of severe hypoglycaemia (where severe hypoglycaemia was defined as an episode of hypoglycaemia associated with neuroglycopaenia and requiring third-party assistance to correct^28^. The first three outcomes were selected based on evidence from previous studies that benchmarking clinical performance with regards to these health outcomes drives quality improvement^2–4,14,18,29^. The severe hypoglycaemia outcome was selected based on clinical importance.

### Risk-modelling

In accordance with a-priori clinical reasoning, patients were stratified by diabetes type to allow for separate analysis of patients with T2DM and T1DM diabetes. For each T2DM and T1DM health outcome, multivariate stepwise regression was performed with p values for variable inclusion and removal set at p < 0.01 and p > 0.05, respectively. Based on a-priori clinical reasoning, patient fasting status at the time of LDL-Ch measurement was forced into the multivariate model for LDL-Ch. Collinearity was identified using variance inflation factor with a cut-off value of 10^30^. Where relevant, the collinear term demonstrating less statistical significance (as determined by the magnitude of the z-statistic) was eliminated from the model. All statistical functions were performed using STATA version 14 (Stata Corp, College Station, Tx, USA).

#### Sensitivity and subgroup analyses

Additional analyses were performed on the multivariate risk models to analyse the impact of outlier values, missing data and treatment. For each of the continuous outcomes, outlier values were determined using the Tukey fence method^31^. Multivariate regression was repeated for each continuous outcome measure with the outliers excluded. The results of this analysis were then compared to the original results.

A second sensitivity analysis assessed the significance of missing data. All numerical study variables with missing rates exceeding 10% were identified. A multiple imputation model was then run using multivariate normal distribution, which accommodates arbitrary missing value patterns using the iterative Markov chain Monte Carlo method. Using predictor variables with high rates of data completeness, 10 datasets were created containing imputed values for the missing data. Regression models were then run separately and combined using the method proposed by Little *et al*.^32^. The results of the multiple imputation analysis were compared to the original results.

Based on a priori clinical reasoning, the effect of treatment on the relationship between risk variables and outcomes was investigated. Patients were stratified according to treatment status (i.e. on insulin vs not on insulin). Regression analyses were subsequently repeated on each treatment subgroup. This was to observe whether stratification by treatment status altered the strength or direction of the association between risk variable and outcome.

### Assessing the impact of risk-adjustment

Unadjusted and risk-adjusted funnel plots were constructed and compared to test the impact of risk-adjustment on performance benchmarking. Unadjusted and risk-adjusted diabetes health outcomes were converted into performance measures of average levels or rates. Average patient measurements per centre were calculated for HbA1c, LDL-Ch and systolic blood pressure. Rates of severe hypoglycaemia were calculated by dividing the total number of patients per centre who had experienced severe hypoglycaemia at least once within the previous 12 months by the total number of patients per centre, and multiplying by 100. Funnel plots were constructed for each unadjusted and risk-adjusted performance measure by plotting centre specific values against sample size (the number of patients per centre for whom data was submitted).

Performance status was determined by the position of a centre relative to 99.8% control limits (three standard deviations, 3SD, above and below the mean). For each performance measure, the magnitude of the average level or rate was considered inversely proportional to clinical performance. For example, lower rates of severe hypoglycaemia indicated better performance. Therefore, centres positioned above the upper control limit (3SD above the mean) were identified as low-performing outliers and centres positioned below the lower control limit (3SD below the mean) were identified as high-performing outliers. All centres within 3SD of the mean were inliers. Centres were therefore classified as (a) inlier (b) low-performing outlier or (c) high-performing outlier for each performance measure.

For each performance measure, unadjusted and risk-adjusted funnel plots were compared to detect changes in performance status resulting from risk-adjustment. Instances where low-performing outliers were reclassified as inliers after risk-adjustment (‘false positives’) and vice versa (‘false negatives’) were recorded. Similarly, false positives and false negatives for high-performance were recorded. Rates of misclassification of outliers were calculated by dividing false positives by the total number of outliers identified by unadjusted funnel plots.

### Data availability

The datasets analysed during the current study are available from the corresponding author on reasonable request.

## Results

### Study population characteristics

Forty-nine centres delivering diabetes care participated in the 2015 Australian National Diabetes Audit (ANDA). This corresponded to 47% of all diabetes centres registered with the National Association of Diabetes Centres (NADC) in 2015. The majority (67%) of participating centres were tertiary diabetes centres, with the remaining 33% being primary care or community-based diabetes clinics. Participating centres were predominantly located in Victoria, New South Wales and Queensland (41%, 27% and 18% respectively), with the remaining centres distributed throughout the other states and territories of Australia. Most (78%) participating centres responded to data query reports by forwarding updated case-record forms after addressing missing and invalid data.

Across the 49 participating centres, data were submitted for 5,183 patients. This included 3,496 patients with type 2 diabetes (T2DM), 1,174 patients with type 1 diabetes (T1DM) and 243 patients with gestational diabetes. 270 patients were of ‘unknown’ or ‘other’ type. For the purpose of this study, only patients with T1DM or T2DM were analysed.

The baseline characteristics of the study population are summarised in Table 1. Overall, patients with T2DM had a median age of 64, median disease duration of 12 years and median BMI of 32 kg/m^2^. Mean values (and standard deviations) for HbA1c (%), LDL-Ch (mmol/L) and systolic blood pressure (mmHg) were 8.2 ± 1.8, 2.1 ± 1.2 and 133 ± 19, respectively. Compared to patients with T2DM, patients with T1DM were, on average, younger (median age 37) and less overweight (median BMI 26 kg/m^2^) but with a longer disease duration (median disease duration 16 years). Patients with T1DM were also half as likely to have been born overseas, and less likely to suffer from complications. Metabolic control was slightly worse in patients with T1DM compared to patients with T2DM patients; on average, HbA1c (%) and LDL-Ch (mmol/L) levels were 0.3 and 0.4 units higher, respectively; but blood pressure was slightly better (systolic blood pressure 9 mmHg lower on average). Patients with T1DM were 3 times more likely than T2DM patients to have experienced at least one severe hypoglycaemic episode within the previous 12 months (13% vs 4%).

**Table 1:** Study population baseline characteristics.

Characteristic	All patients (n = 4,670)	Type 2 patients (n = 3,496)	Type 1 patients (n = 1,174)
**Demographics**			
Age (years): median (IQR)	60 (46–69)	64 (55–72)	37 (25–52)
Sex			
Male: n (%)	2,399 (52%)	1,862 (54%)	537 (47%)
Female: n (%)	2,203 (48%)	1,592 (46%)	611 (53%)
Country of birth			
Australia: n (%)	2,709 (65%)	1,820 (59%)	889 (83%)
Overseas: n (%)	1,428 (35%)	1,240 (41%)	188 (17%)
**Body mass index**			
Underweight (<18.5 kg/m^2^): n (%)	26 (<1%)	4 (<1%)	22 (2%)
Healthy weight (18.5–24.9 kg/m^2^): n (%)	734 (18%)	314 (10%)	420 (42%)
Overweight (25–29.9 kg/m^2^): n (%)	1,170 (28%)	855 (27%)	315 (31%)
Obese (30–39.9 kg/m^2^): n (%)	1,672 (40%)	1,446 (46%)	226 (22%)
Morbidly obese (≥40 kg/m^2^): n (%)	535 (13%)	509 (16%)	26 (3%)
**Disease characteristics**			
Diabetes duration (years): median (IQR)	13 (7–20)	12 (6–20)	16 (8–27)
Severity			
DCSI score 0: n (%)	1,608 (34%)	998 (29%)	610 (52%)
DCSI score 1–2: n (%)	1,619 (35%)	1,294 (37%)	325 (28%)
DCSI score 3–4: n (%)	845 (18%)	721 (21%)	124 (11%)
DCSI score ≥ 5: n (%)	598 (13%)	483 (14%)	115 (10%)
**Smoking status**			
Never: n (%)	2,152 (54%)	1,515 (51%)	637 (62%)
Ever (current or past): n (%)	1,859 (46%)	1,461 (49%)	398 (38%)
Current: n (%)	568 (14%)	396 (13%)	172 (17%)
Past: n (%)	1,291 (32%)	1,065 (36%)	226 (22%)
**Treatment**			
Insulin: n (%)	3,280 (70%)	2,120 (61%)	1,160 (99%)
Lipid-lowering medication(s): n (%)	2,862 (62%)	2,534 (73%)	328 (28%)
Antihypertensive medication(s): n (%)	2,903 (64%)	2,583 (76%)	320 (28%)
**Health outcomes**			
HbA1c (%): mean ± SD	8.3 ± 1.8	8.2 ± 1.8	8.5 ± 1.8
LDL-Ch (mmol/L): mean ± SD	2.2 ± 1.2	2.1 ± 1.2	2.5 ± 0.9
SBP (mmHg): mean ± SD	131 ± 19	133 ± 19	124 ± 17
Severe hypo (≥1 episode in previous year): n (%)	269 (6%)	125 (4%)	144 (13%)

Abbreviations: DCSI = diabetes complications severity index, HbA1c = glycated haemoglobin, hypo = hypoglycaemia, IQR = interquartile range, LDL-Ch = low density lipoprotein cholesterol, SBP = systolic blood pressure, SD = standard deviation.

Note: patients with missing data were excluded from percentage calculations.

### Risk models for diabetes health outcomes

Several non-care related patient factors were significantly associated with diabetes health outcomes. The impact of patient factors varied across the different T1DM and T2DM health outcomes. Consequently, eight unique risk models were produced (Table 2).

**Table 2:** Multivariate risk-adjustment of diabetes outcomes.

Population	Outcome	Model	Coef.	CI	p-value	aR^2^
T2DM	HbA1c	Female sex (ref = male)	0.29	(0.13, 0.44)	<0.001	0.044
		Age (per 10 years)	−0.30	−0.36, −0.23)	<0.001	
		Duration (per 10 years)	0.22	(0.13, 0.31)	<0.001	
		Severity (ref = DSCI score 0)				
		DCSI score 1-2	0.41	(0.22, 0.60)	<0.001	
		DCSI score 3-4	0.34	(0.11, 0.56)	0.003	
		DCSI score ≥5	0.31	(0.05, 0.57)	0.019	
		Ever smoker (ref = never smoker)	0.21	(0.07, 0.36)	0.005	
	LDL-Ch	Fasting status† (ref = non-fasting)	−0.01	(−0.15, 0.13)	0.914	0.078
		Age (per 10 years)*	−0.13	(−0.17, −0.09)	<0.001	
		Duration (per 10 years)*	−0.08	(−0.13, −0.03)	0.002	
		Severity (ref = DSCI score 0)				
		DCSI score 1-2	−0.16	(−0.28, −0.05)	<0.001	
		DCSI score 3-4	−0.27	(−0.40, −0.13)	<0.001	
		DCSI score ≥5	−0.31	(−0.46, −0.15)	<0.001	
	SBP	Age (per 10 years)*	2.24	(1.68, 2.79)	<0.001	0.042
		High BMI (ref = healthy BMI)				
		Overweight	2.85	(0.48, 5.52)	0.018	
		Obese	5.13	(2.88, 7.37)	<0.001	
		Morbidly obese	9.24	(6.63, 11.85)	<0.001	
		Severity (ref = DSCI score 0)				
		DCSI score 1-2	2.09	(0.45, 3.73)	0.012	
		DCSI score 3-4	3.02	(1.10, 4.94)	0.002	
		DCSI score ≥5	2.21	(0.01, 4.40)	0.048	
T1DM	HbA1c	Age (per 10 years)*	−0.20	(−0.28, −0.12)	<0.001	0.048
		Underweight (ref = healthy BMI)	1.73	(0.91, 2.55)	<0.001	
		Severity (ref = DSCI score 0)				
		DCSI score 1-2	0.33	(0.03, 0.62)	0.032	
		DCSI score 3-4	0.21	(−0.23, 0.65)	0.344	
		DCSI score ≥5	0.61	(0.14, 1.08)	0.010	
		Ever smoker (ref = never smoker)	0.33	(0.08, 0.59)	0.011	
	LDL-Ch	Fasting status† (ref = non-fasting)	−0.08	(−0.27, 0.12)	0.442	0.051
		Duration (per 10 years)*	−0.14	(−0.19, −0.09)	<0.001	
	SBP	Male sex (ref = female)	6.06	(3.99, 8.12)	<0.001	0.210
		Age (per 10 years)*	2.51	(1.70, 3.31)	<0.001	
		High BMI (ref = healthy BMI)				
		Overweight	1.65	(−0.73, 4.04)	0.173	
		Obese	6.42	(3.76, 9.07)	<0.001	
		Morbidly obese	7.28	(1.03, 13.54)	0.023	
		Duration (per 10 years)*	2.01	(1.08, 2.94)	<0.001	
**Population**	**Outcome**	**Model**	**OR**	**CIs**	**p-value**	**ROC**
T2DM	Severe hypo	Duration (per 10 years)*	1.54	(1.27, 1.86)	<0.001	0.613
T1DM	Severe hypo	Duration (per 10 years)*	1.39	(1.23, 1.58)	<0.001	0.678
		High BMI (ref = healthy BMI)				
		Overweight	0.84	(0.54, 1.31)	0.446	
		Obese	0.47	(0.27, 0.83)	0.009	
		Morbidly obese	0.39	(0.08, 1.75)	0.217	
		Ever smoker (ref = never smoker)	1.96	(1.33, 2.89)	0.001	

Abbreviations: aR^2^ = adjusted R^2^, CI = confidence interval, Coef. = coefficient, DCSI = diabetes complications severity index, HbA1c = glycated haemoglobin (%), hypo = hypoglycaemia (≥ 1 episodes in previous 12 months), LDL-Ch = low density lipoprotein cholesterol (mmol/L), OR = odds ratio, ROC = receiver operator characteristic (area under curve), SBP = systolic blood pressure (mmHg), T1DM = type 1 diabetes mellitus, T2DM = type 2 diabetes mellitus.

*Coefficient calculated for age/duration (per 10 years) indicates average change in outcome associated with a 10-year increase in age/duration.

^†^Fasting status was fixed into the multivariate risk model based on a priori clinical reasoning.

#### HbA1c

Among patients with T2DM, factors associated with higher HbA1c (%) levels were younger age, female sex, longer disease duration, positive smoking history and the presence of complications (DCSI ≥ 1). Compared to the reference group (patients with no complications), HbA1c levels were 0.41 units higher (95% CI: 0.22 to 0.60, p < 0.001) in patients with a DCSI score of 1–2.

Among patients with T1DM, factors associated with higher HbA1c levels were younger age, underweight (BMI < 18.5 kg/m^2^), worsening disease severity and positive smoking history. The greatest magnitude of effect was observed for underweight, which was associated with HbA1c levels that were 1.73 units higher (95% CI: 0.91 to 2.55, p < 0.001) compared to those with a healthy BMI.

#### LDL-Ch

Among patients with T2DM, factors associated with higher LDL-Ch (mmol/L) levels were younger age, shorter disease duration and lower disease severity. Compared to those with no complications, those with the most severe disease (DCSI ≥ 5) had lower average LDL-Ch levels by 0.31 mmol/L (95% CI: −0.46 to −0.15, p < 0.001). Each 10-year increase in age was associated with a decrease in LDL-Ch level of 0.13 units (95% CI: −0.17 to −0.09, p < 0.001).

Among patients with T1DM, only duration of disease was significantly associated with LDL-Ch levels. Each 10-year increase in disease duration was associated with a decrease in LDL-Ch levels of 0.14 mmol/L (95% CI −0.19 to −0.09, p < 0.001).

#### Systolic blood pressure

Among patients with T2DM, factors associated with higher systolic blood pressure were older age, higher BMI and greater disease severity. In particular, obesity and morbid obesity was associated with higher systolic blood pressures, by 5.13 (95% CI: 2.88 to 7.37, p < 0.001) and 9.24 (95% CI: 6.63 to 11.85, p < 0.001) units respectively.

Among patients with T1DM, factors associated with higher systolic blood pressure were male sex, older age, higher BMI and longer duration of disease. Male sex and obesity accounted for the greatest effect on systolic blood pressure. Males had systolic blood pressure readings 6.06 units higher on average compared to females (95% CI: 3.99 to 8.12, p < 0.001). Compared to patients with a healthy BMI, patients with obesity and morbid obesity had higher systolic blood pressure measurements by 6.42 (95% CI: 3.76 to 9.07, p < 0.001) and 7.28 units (95% CI: 1.03 to 13.54, p = 0.023), respectively.

#### Severe hypoglycaemia

Among patients with T2DM, only disease duration was significantly associated with the odds of experiencing severe hypoglycaemia. Each 10-year increase in age was associated with a 54% increase in odds of severe hypoglycaemia (95% CI 1.27 to 1.86, p < 0.001).

Among patients with T1DM, the odds of experiencing severe hypoglycaemia were associated with disease duration, high BMI and smoking history. Each 10-year increase in disease duration was associated with a 39% increase in odds of severe hypoglycaemia (95% CI: 1.23 to 1.58, p < 0.001). Compared to patients with a healthy BMI, obese patients had a 53% reduction in odds of severe hypoglycaemia (95% CI: 0.27 to 0.83, p = 0.009). Compared to never smoking, ever smoking was associated with a 96% increase in odds of experiencing severe hypoglycaemia (95% CI: 1.33 to 2.89, p = 0.001).

#### Sensitivity and subgroup analyses

Additional analyses were performed to evaluate the impact of outlier values, missing data and treatment.

Exclusion of outlier values from the multivariate analysis of glycaemic control resulted in elimination of underweight from the T1DM risk model, and elimination of sex from the T2DM risk model. Exclusion of outlier values from the multivariate analysis of LDL-Ch and systolic blood pressure did not significantly change results.

Multiple imputation of numerical variables with missing data rates exceeding 10% (i.e. LDL-Ch, HbA1c and BMI) did not significantly change results.

When associations between risk variables and outcomes were analysed across treatment subgroups, two differences were observed. Stratification of T2DM patients by insulin status reversed the effects of both disease duration and disease severity on glycaemic control. Shorter duration and reduced severity of disease were associated with poorer glycaemic control in insulin-taking T2DM patients.

### Impact of risk-adjustment on performance benchmarking

**Figure 1:**
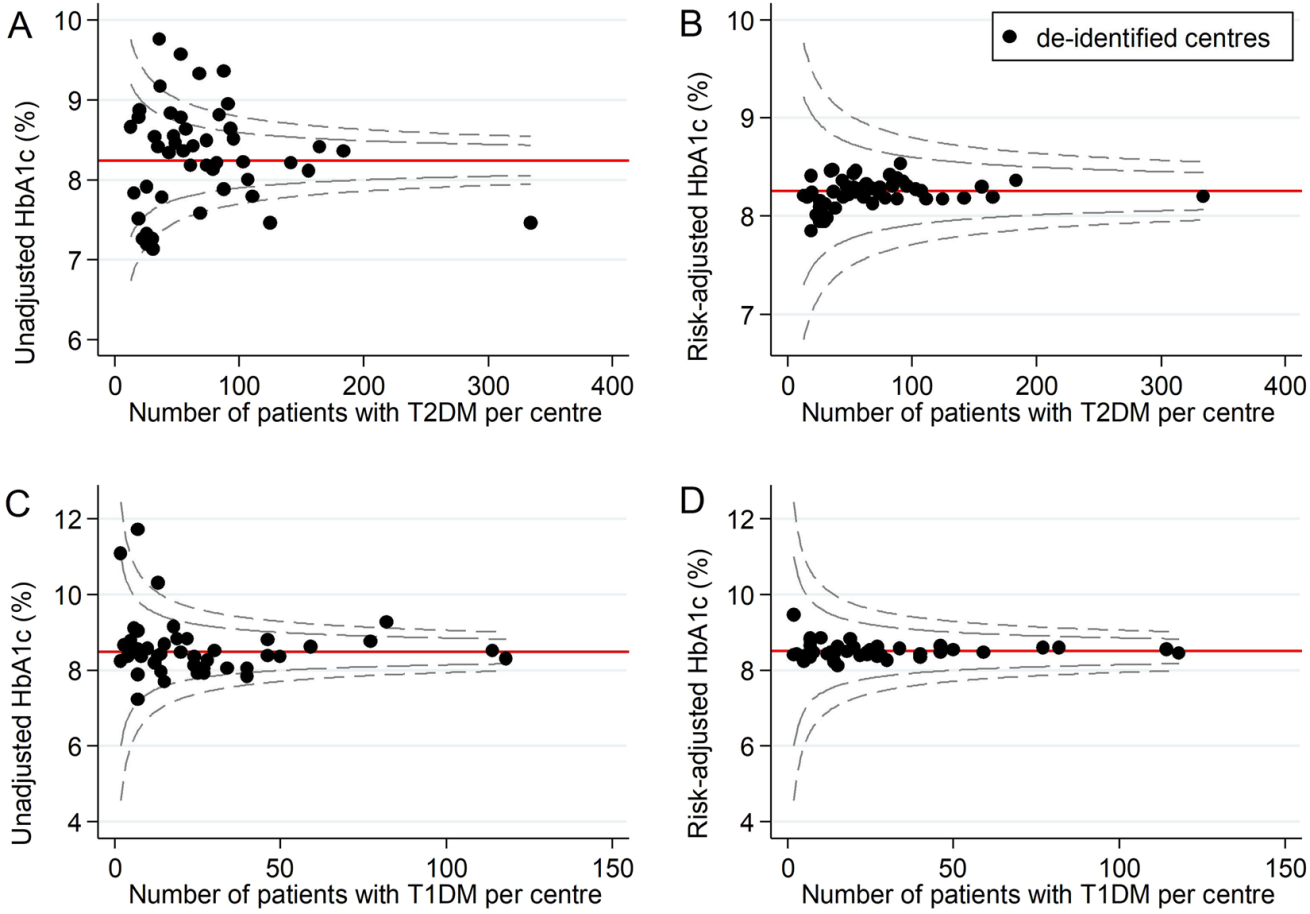
Unadjusted and risk-adjusted funnel plots of mean HbA1c (%) levels. These funnel plots compare unadjusted and risk-adjusted clinical performance with regards to mean HbA1 (%) levels. 99.8% (outer limits) and 95% (inner limits) control limits are shown. (**A**) Mean unadjusted HbA1c (%) in patients with T2DM and (**B**) Mean risk-adjusted HbA1c (%) in patients with T2DM (adjusted for age, sex, BMI, duration and severity; (**C**) Mean unadjusted HbA1c (%) in patients with T1DM and (**D**) Mean risk-adjusted HbA1c (%) in patients with T1DM (adjusted for age, BMI, severity and smoking history).

**Figure 2:**
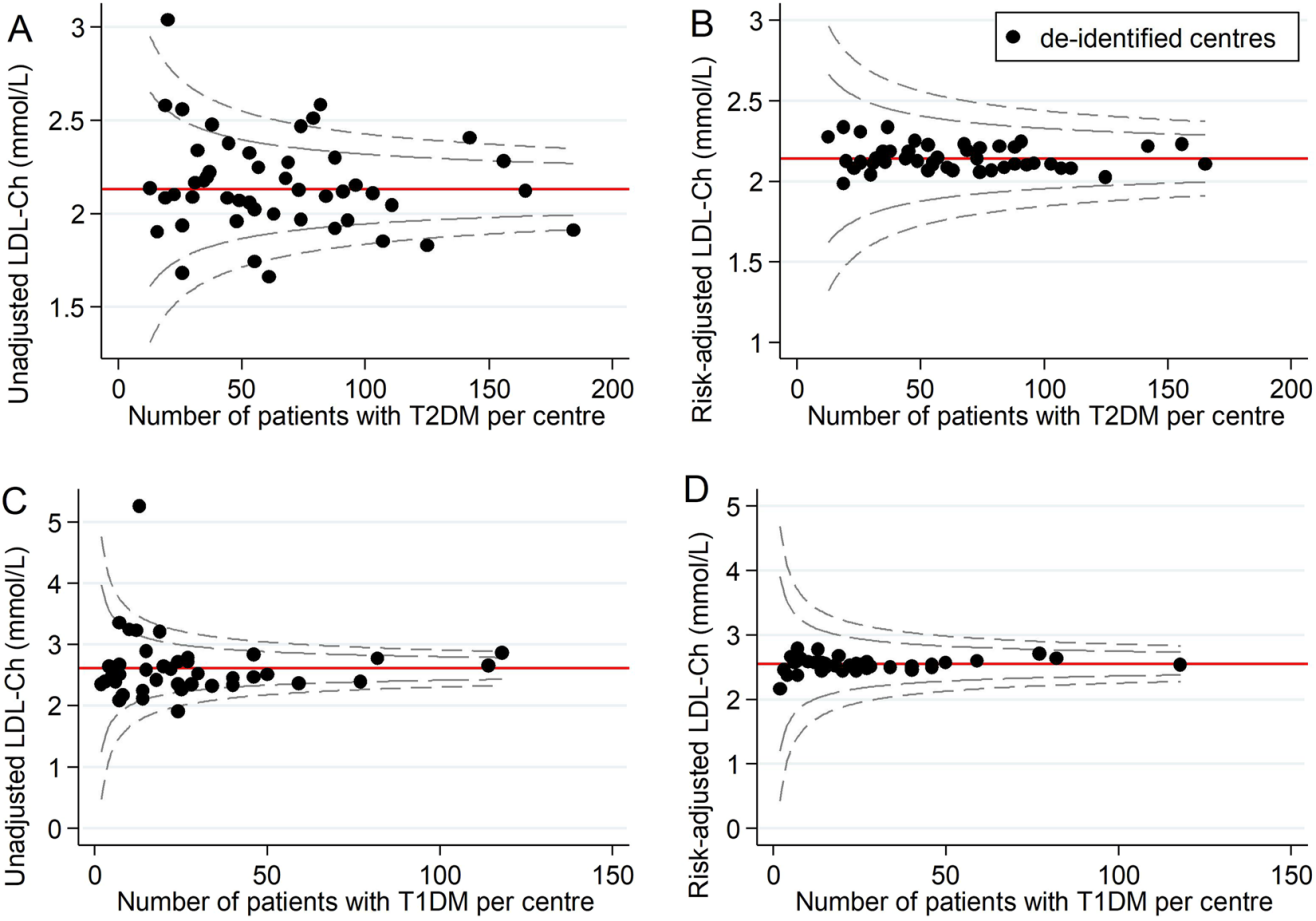
Unadjusted and risk-adjusted funnel plots of mean LDL-Ch (mmol/L) levels. These funnel plots compare unadjusted and risk-adjusted clinical performance with regards to mean LDL-Ch (mmol/L) levels. 99.8% (outer limits) and 95% (inner limits) control limits are shown. (**A**) Mean unadjusted LDL-Ch (mmol/L) in patients with T2DM and (**B**) Mean risk-adjusted LDL-Ch (mmol/L) in patients with T2DM (adjusted for age, disease duration, disease severity, and fasting status); (**C**) Mean unadjusted LDL-Ch (mmol/L) in patients with T1DM and (**D**) Mean risk-adjusted LDL-Ch (mmol/L) in patients with T1DM (adjusted for disease duration and fasting status).

**Figure 3:**
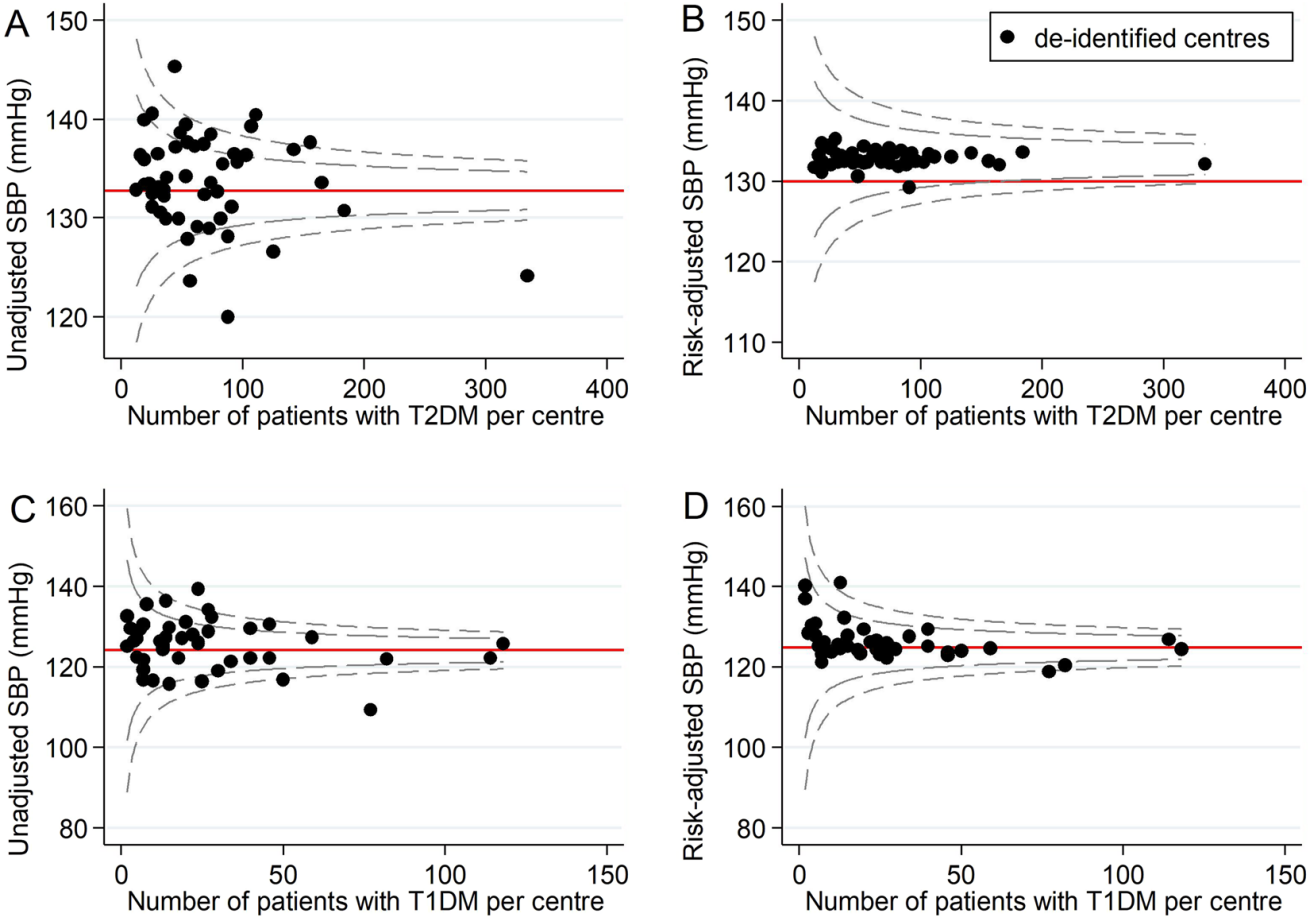
Unadjusted and risk-adjusted funnel plots of mean systolic blood pressure (mmHg) measurements. These funnel plots compare unadjusted and risk-adjusted clinical performance with regards to mean systolic blood pressure (SBP) measurements (mmHg). 99.8% (outer limits) and 95% (inner limits) control limits are shown. (**A**) Mean unadjusted SBP (mmHg) in patients with T2DM and (**B**) Mean risk-adjusted SBP (mmHg) in patients with T2DM (adjusted for age, BMI and disease severity); (**C**) Mean unadjusted SBP (mmHg) in patients with T1DM and (**D**) Mean risk-adjusted SBP (mmHg) in patients with T1DM (adjusted for sex, age, BMI and disease duration).

**Figure 4:**
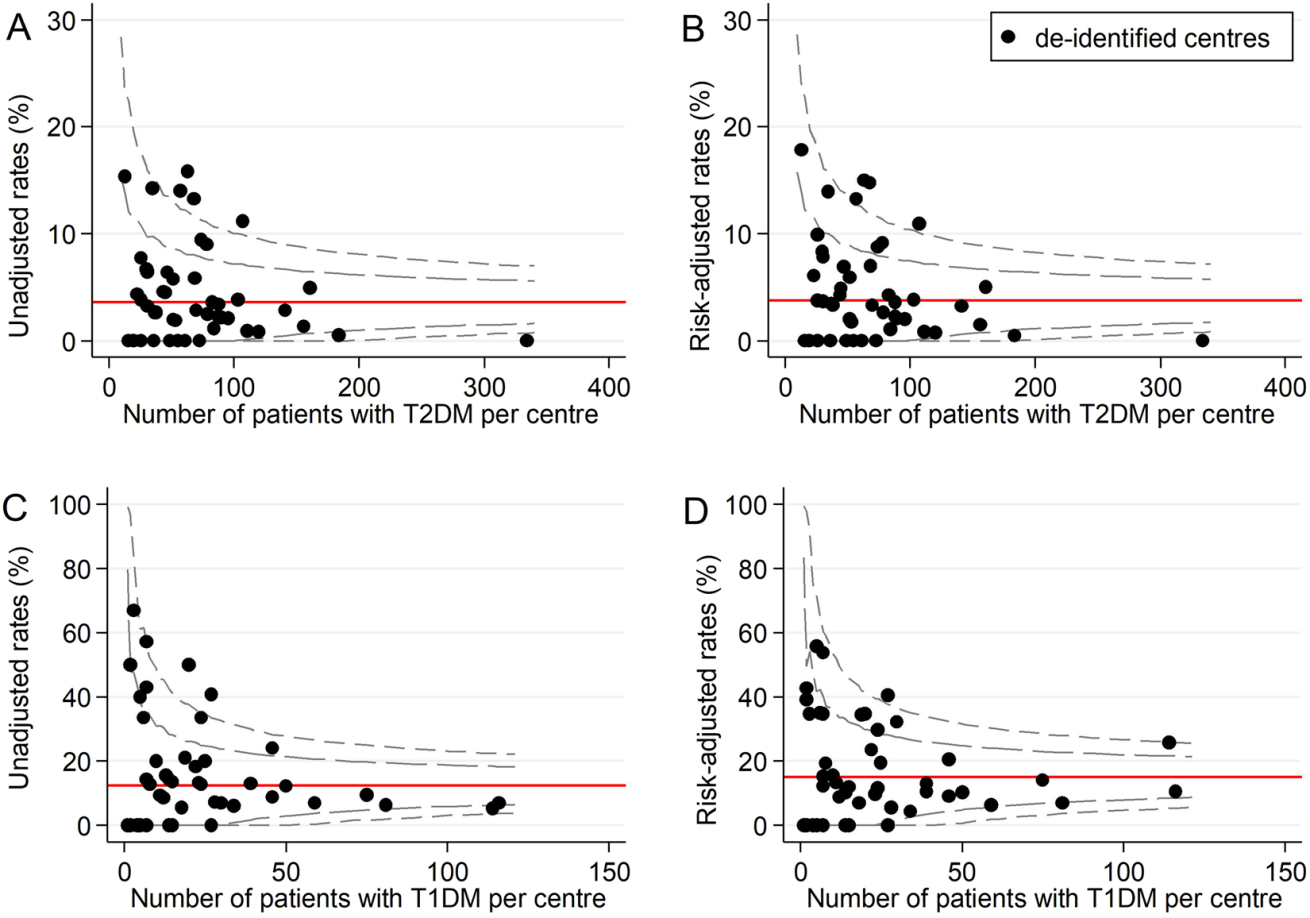
Unadjusted and risk-adjusted rates of severe hypoglycaemia (%). These funnel plots compare unadjusted and risk-adjusted clinical performance with regards to rates of severe hypoglycaemia (proportion of patients who experienced one or more episodes of severe hypoglycaemia within the previous 12 months). 99.8% (outer limits) and 95% (inner limits) control limits are shown. (**A**) Unadjusted rates (%) in patients with T2DM and (**B**) Risk-adjusted rates (%) in patients with T2DM (adjusted for disease duration); (**C**) Unadjusted rates (%) in patients with T1DM and (**D**) Risk-adjusted rates (%) in patients with T1DM (adjusted for disease duration, BMI and smoking history).

When the risk models were applied to funnel plots, we observed changes to the number of identified low-performing or high-performing outliers relative to 99.8% control limits (i.e. 3 SD above or below the mean). Across all diabetes health outcomes, unadjusted funnel plots identified 27 low-performing outliers and 15 high-performing outliers. After risk-adjustment, 22 (81%) low-performing outliers and 13 (87%) high-performing outliers became inliers (false positives). Additionally, one inlier became a low-performing outlier (false negative) (Figs 1–4).

#### T2DM health outcomes

Across all T2DM health outcomes, unadjusted funnel plots identified 18 low-performing outliers and 12 high-performing outliers. Of these, 14 (78%) low-performing outliers and 11 (92%) high-performing outliers were false positives. For each outcome, the number of false positives for low-performance ranged from 0 to 6 and the number of false positives for high-performance ranged from 0 to 4. There were no outlier false positives for the severe hypoglycaemia outcome, and no outlier false negatives for any of the T2DM health outcomes.

#### T1DM health outcomes

Across all T1DM health outcomes, unadjusted funnel plots identified 9 low performing outliers and 3 high-performing outliers. Of these, 8 (89%) low-performing outliers and 2 (67%) high-performing outliers were false positives. For each outcome, the number of false positives for low-performance ranged from 1 to 3 and the number of false positives for high-performance ranged from 0 to 2. There was one outlier false negative for low-performance for systolic blood pressure (Table 3).

**Table 3:** Impact of risk-adjustment on performance status.

Diabetes health outcome		Low-performing outliers		High-performing outliers	
		Total	False positives	Total	False positives
T2DM	HbA1c	6	6 (100%)	4	4 (100%)
	LDL-Ch	4	4 (100%)	3	3 (100%)
	SBP	4	4 (100%)	4	4 (100%)
	Severe hypo	4		1	
	Total	18	14 (78%)	12	11 (92%)
T1DM	HbA1c	3	3 (100%)		
	LDL-Ch	1	1 (100%)	1	1 (100%)
	SBP	2	2 (100%)	2	1 (50%)
	Severe hypo	3	2 (67%)		
	Total	9	8 (89%)	3	2 (67%)
Overall	Total	27	22 (81%)	15	13 (87%)

Abbreviations: HbA1c = glycated haemoglobin, hypo = hypoglycaemia, LDL-Ch = low-density lipoprotein cholesterol, SBP = systolic blood pressure, T1DM = type 1 diabetes mellitus, T2DM = type 2 diabetes mellitus.

## Discussion

This is the first study to analyse the impact of risk-adjustment on clinical performance benchmarking across Australian diabetes centres. We have demonstrated that risk-adjusting for non-care related patient factors (age, sex, disease duration and severity, body mass index and smoking history) significantly impacts on performance benchmarking with regards to the identification of low-performing and high-performing outliers. Our study provides compelling evidence to support risk-adjustment of diabetes health outcomes to facilitate fairer, more accurate benchmarking of clinical performance in diabetes care.

Risk-adjustment of diabetes health outcomes resulted in a significant reduction in low-performing outliers, or ‘false positives’. False positives are associated with several adverse outcomes. For example, negative representations of performance may lower morale and create unwarranted anxiety in centres that are actually performing at a satisfactory level relative to their peers^33^. Furthermore, false positives may result in the misdirection of resources towards areas of apparent underperformance^34^. Given the finite nature of healthcare funding, this may be at the expense of areas that would be more likely to benefit from additional funding. Risk-adjustment protects against these adverse outcomes by minimising false alarms for low performance.

Risk-adjustment also reduced ‘false negatives’ for low performance by enabling the identification of a previously undetected low-performing outlier. False negatives may engender inappropriate complacency in centres that are underperforming relative to peer centres^33^. With risk-adjusted feedback, centres participating in benchmarking are more likely to identify and address clinical care issues that fall under the influence of healthcare intervention. Furthermore, we observed that risk-adjustment reduced the number of identified high performing outliers. Risk-adjustment therefore attenuates the complacency and consequent demotivation to strive for improvement that may result from inaccurate representations of high performance^33^.

Of note, risk-adjustment removed most variation in clinical performance with regards to HbA1c, LDL-Ch and systolic blood pressure in both patients with T2DM and T1DM. This suggests relative consistency in the quality of T2DM and T1DM care delivered across Australian diabetes centres participating in the 2015 ANDA. However, significant variation in clinical performance was observed even after risk-adjustment for severe hypoglycaemia in patients with T2DM. This may reflect that severe hypoglycaemia rates are not typically reported as indicators of clinical performance^10,35^. Consequently, clinicians may neglect this clinical care issue in order to focus on optimising the health outcomes that are likely to influence benchmarking (‘tunnel vision’ phenomenon)^33^. Ongoing benchmarking of severe hypoglycaemia may assist to combat this largely ‘unrecognised healthcare burden’^36^ by directing attention to this clinical care issue and incentivising targeted preventative strategies.

There are several strengths to our study. Firstly, our use of data from a nationwide diabetes benchmarking initiative is appropriate in the context of our primary objective: to inform benchmarking of clinical performance across Australian diabetes centres. Our study cohort is highly representative of the patients and centres who stand to benefit from changes to current practice in benchmarking. Furthermore, we are confident in the reliability of our dataset given that the ANDA dataset has been clinically validated during previous nationwide audits and subject to multiple reviews and additions. All data items were based on standardised, objective definitions to minimise the risk of measurement bias.

Another key strength of this study is that the risk models were tailored to diabetes type. By contrast, previous studies of risk-modelling for diabetes health outcomes have not distinguished between patients with T2DM and T1DM^20,22,24,25,37,38^. Our decision to stratify patients by diabetes type enabled us to identify differences in risk models for patients with T2DM compared to T1DM. For example, duration of diabetes was a risk factor for poorer glycaemic control in patients with T2DM but not in patients with T1DM. This is not surprising given the differences in the pathogenesis and natural history between T2DM and T1DM. Indeed, T2DM is characterised by progressive β-cell dysfunction leading to progressive loss of insulin secretion^39^. A worsening of glycaemic control may therefore be anticipated in patients with increasing duration of T2DM. Our findings suggest that stratification by diabetes type avoids inappropriate generalisations and improves the accuracy and validity of risk modelling.

Our sensitivity analyses indicated that our results were minimally impacted by missing data and outlier values. Furthermore, most associations between patient risk factors and diabetes health outcomes were unchanged when analysed across treatment subgroups. This suggests that the identified risk factors were unrelated to treatment and therefore suitable for inclusion in the risk models. Only two changes were observed during the subgroup analyses: increasing severity and duration of diabetes were each associated with lower HbA1c levels in patients with T2DM taking insulin. Duration and severity of disease are likely on the causal pathway to intensification of insulin treatment in patients with T2DM^39^. This could account for a lowering of HbA1c levels.

Our study is subject to limitations. Our risk models only included variables that were able to be retrieved/calculated from the minimal dataset collected by the audit activity. We were unable to adjust for unmeasured confounders including socio-economic status. There was significant variation in statistical performance between our risk models, with adjusted R^2^ values ranging from 4.2% to 21%. This variation may reflect the varying degree to which unmeasured patient risk factors impact on different study outcomes. Future studies should consider testing for the impact of additional non-care related patient factors on benchmarking of clinical performance.

In conclusion, our study demonstrates that risk-adjustment for non-care related patient risk factors significantly impacts performance benchmarking in diabetes care by reducing false positives for outlier performance. We recommend that risk-adjustment be performed on diabetes health outcomes for benchmarking to reduce misclassification of performance and provide more accurate feedback to inform subsequent quality improvement activity. We also recommend that clinicians focus on prevention of severe hypoglycaemia in patients with T2DM.

## References

[1] Guldberg, T. L., Lauritzen, T., Kristensen, J. K. & Vedsted, P. The effect of feedback to general practitioners on quality of care for people with type 2 diabetes: a systematic review of the literature. *BMC Fam*. *Pract*. **10**, 10.1186/1471-2296-10-30 (2009). 10.1186/1471-2296-10-30 PMC2690581 19419548

[2] Kiefe CI (2001). Improving quality improvement using achievable benchmarks for physician feedback: a randomized controlled trial. JAMA..

[3] Debacker N (2008). Organization of a quality-assurance project in all Belgian multidisciplinary diabetes centres treating insulin-treated diabetes patients: 5 years’ experience. Diabetic Med..

[4] Hermans MP (2013). Benchmarking is associated with improved quality of care in type 2 diabetes: the OPTIMISE randomized, controlled trial. Diabetes Care..

[5] McNeil, J. Registry Science Handbook. *Monash University*, https://www.monash.edu/medicine/sphpm/registries/resources (2013).

[6] Gottwald, M. & Lansdown, G. Evaluating quality care through audit in *Clinical governance: improving the quality of healthcare for patients and service users* (ed. Gottwald, M. & Lansdown, G.) 165–187 (McGraw-Hill Education, 2014).

[7] Wilcox N, McNeil JJ (2016). Clinical quality registries have the potential to drive improvements in the appropriateness of care. Med J. Aust..

[8] Kerr EA, Krein SL, Vijan S, Hofer TP, Hayward RA (2001). Avoiding pitfalls in chronic disease quality measurement: a case for the next generation of technical quality measures. Am. J. Manag. Care..

[9] Mant J (2001). Process versus outcome indicators in the assessment of quality of health care. Int. J. Qual. Health Care..

[10] Sidorenkov G, Haaijer-Ruskamp FM, de Zeeuw D, Bilo H, Denig P (2011). Review: relation between quality-of-care indicators for diabetes and patient outcomes: a systematic literature review. Med. Care Res. Rev..

[11] AHRQ. Types of quality measures. *Agency for Healthcare Research and Quality*, https://www.ahrq.gov/professionals/quality-patient-safety/talkingquality/create/types.html (2011).

[12] Iezzoni, L. Risk adjustment for performance measurement in *Performance measurement for health system improvement* (eds Smith, P., Mossialos, E., Papanocolas, I. & Leatherman, S.) 251–285 (Cambridge University Press, 2009).

[13] MCHRI. ANDA-AQCA 2015: Australian National Diabetes Audit, Final Report. 231 p. (MCHRI, 2015).

[14] Hunt JS (2009). The impact of a physician-directed health information technology system on diabetes outcomes in primary care: a pre- and post-implementation study. Inform. Prim. Care..

[15] Fleming BB (2001). The Diabetes Quality Improvement Project: moving science into health policy to gain an edge on the diabetes epidemic. Diabetes Care..

[16] Socialstyrelsen. Quality and efficiency of diabetes care in sweden – national performance assessment 2011. *Socialstyrelsen*, http://www.socialstyrelsen.se/publikationer2014/2014-3-18 (2014).

[17] NICE. NICE quality and outcomes framework indicator: diabetes mellitus. *The National Institute for Health and Care Excellence*, https://www.nice.org.uk/standards-and-.

[18] Rossi MC (2008). Baseline quality-of-care data from a quality-improvement program implemented by a network of diabetes outpatient clinics. Diabetes Care..

[19] Krumholz HM (2006). Standards for statistical models used for public reporting of health outcomes: an American Heart Association scientific statement from the Quality of Care and Outcomes Research Interdisciplinary Writing Group. Circulation..

[20] Safford, M. M. *et al*. Patient complexity in quality comparisons for glycemic control: an observational study. *Implement*. *Sci*. **4****(****1****)**, 10.1186/1748-5908-4-2 (2009). 10.1186/1748-5908-4-2 PMC2632611 19126229

[21] Calsbeek H, Markhorst J, Voerman GE, Braspenning J (2016). Case-mix adjustment for diabetes indicators: a systematic review. Am. J. Manag. Care..

[22] Ta S (2009). Addressing physician concerns about performance profiling: experience with a local veterans affairs quality evaluation program. Am. J. Med. Qual..

[23] Kaplan SH, Griffith JL, Price LL, Pawlson LG, Greenfield S (2009). Improving the reliability of physician performance assessment: identifying the ‘physician effect’ on quality and creating composite measures. Med. Care..

[24] Maney M, Tseng CL, Safford MM, Miller DR, Pogach LM (2007). Impact of self-reported patient characteristics upon assessment of glycemic control in the veterans health administration. Diabetes Care..

[25] Zhang Q (2000). Performance status of health care facilities changes with risk adjustment of HbA1c. Diabetes Care..

[26] d’Emden MC, Shaw JE, Jones GR, Cheung NW (2015). Guidance concerning the use of glycated haemoglobin (HbA1c) for the diagnosis of diabetes mellitus. Med. J. Aust..

[27] Young BA (2008). Diabetes complications severity index and risk of mortality, hospitalization, and healthcare utilization. Am. J. Manag. Care..

[28] Seaquist ER (2013). Hypoglycemia and diabetes: a report of a workgroup of the American Diabetes Association and the Endocrine Society. Diabetes Care..

[29] Goderis G (2010). Start improving the quality of care for people with type 2 diabetes through a general practice support program: a cluster randomized trial. Diabetes Res. Clin. Pract..

[30] Craney TA, Surles JG (2002). Model-dependent variance inflation factor cutoff values. Qual. Eng..

[31] Sullivan, L. & La Morte, W. Interquartile range (IQR). *Boston University School of Public Health*, http://sphweb.bumc.bu.edu/otlt/mph-modules/bs/bs704_summarizingdata/bs704_summarizingdata7.html (2016).

[32] Little, R. J. A. & Rubin, D. B. Statistical analysis with missing data (Wiley, 2014).

[33] Mannion, R. Measuring hospital quality and performance. *The Quarterly RACMA*, http://www.racma.edu.au/index.php?option=com_content&view=article&id=505:measuring-hospital-quality-and-performance&catid=145:the-quarterly-2012&Itemid=256 (2012)

[34] Eijkennaar F (2013). Key issues in the design of pay for performance programs. Eur. J. Health Econ..

[35] Calsbeek H, Ketelaar NABM, Faber MJ, Wensing M, Braspenning J (2013). Performance measurements in diabetes care: the complex task of selecting quality indicators. Int. J. Qual. Health Care..

[36] Villani M (2016). Utilisation of emergency medical services for severe hypoglycaemia: an unrecognised health care burden. J. Diabetes Complications..

[37] Greenfield S, Kaplan SH, Kahn R, Ninomiya J, Griffith JL (2002). Profiling care provided by different groups of physicians: effects of patient case-mix (bias) and physician-level clustering on quality assessment results. Ann. Intern. Med..

[38] Krein SL, Hofer TP, Kerr EA, Hayward RA (2002). Whom should we profile? Examining diabetes care practice variation among primary care providers, provider groups, and health care facilities. Health Serv. Res..

[39] Wong, J. & Yue, D. Starting insulin treatment in type 2 diabetes. *Aust*. *Prescriber*. **27**, 10.18773/austprescr.2004.075 (2004).

